# A Study on the Materials Used in the Ancient Architectural Paintings from the Qing Dynasty Tibetan Buddhist Monastery of Puren, China

**DOI:** 10.3390/ma16196404

**Published:** 2023-09-26

**Authors:** Gele Teri, Kezhu Han, Dan Huang, Yanli Li, Yuxiao Tian, Xiaolian Chao, Zhihui Jia, Peng Fu, Yuhu Li

**Affiliations:** 1 Engineering Research Center of Historical Cultural Heritage Conservation, Ministry of Education, School of Materials Science and Engineering, Shaanxi Normal University, Xi’an 710119, China; terigelesnnu@163.com (G.T.); hankekezhu@126.com (K.H.); hdansnnu@163.com (D.H.); lyl01211106@snnu.edu.cn (Y.L.); tianyuxiao_tyx@163.com (Y.T.); chaoxl@snnu.edu.cn (X.C.); 2 Shaanxi Institute for the Preservation of Culture Heritage, Xi’an 710075, China

**Keywords:** pigment analyses, building conservation, adhesive

## Abstract

Situated in the village of Lama Temple on the eastern bank of the Wulie River in Chengde, Puren Temple stands as one of the few remaining royal temples of great importance from the Kangxi era (1662–1722 AD). This ancient edifice has greatly contributed to the advancement of our comprehension regarding the art of royal temple painting. The present study undertakes a comprehensive analysis and identification of nine samples obtained from the beams and ceiling paintings within the main hall of Puren Temple. Furthermore, a systematic examination of their mineral pigments and adhesives is conducted. The findings from polarized light microscopy (PLM), energy-type X-ray fluorescence spectrometer (ED-XRF), micro-Raman spectroscopy (m-RS), and X-ray diffractometer (XRD) analyses reveal that the pigments present in the main hall beams of Puren Temple are cinnabar, lead white, lapis lazuli, and lime green, while the pigments in the ceiling paintings consist of cinnabar, staghorn, lead white, lapis lazuli, and lime green. The use of animal glue as a binder for these pigments on both the main hall beams and ceiling paintings is confirmed via pyrolysis-gas chromatography–mass spectrometry (Py-Gc/Ms) results. These findings hold significant implications for the future restoration of Puren Temple, as they provide valuable guidance for the selection of appropriate restoration materials.

## 1. Introduction

**Figure 1:**
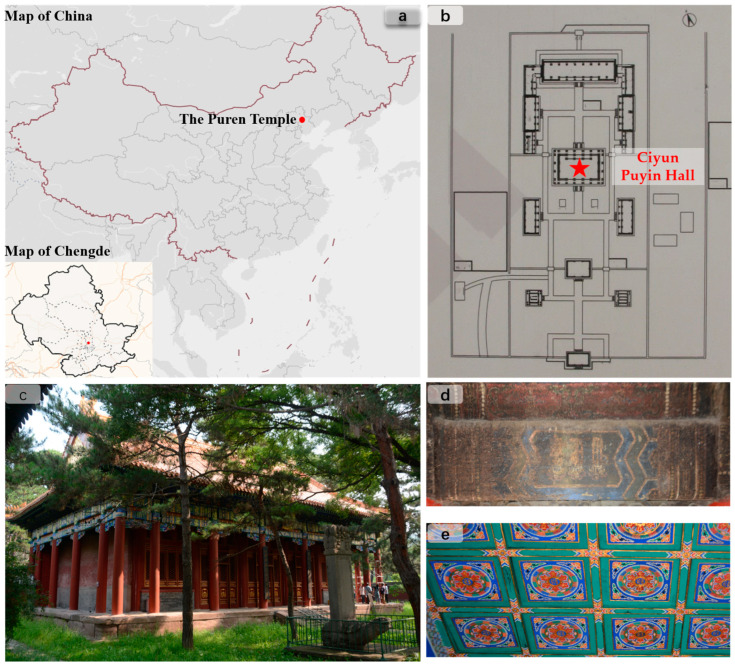
Puren Temple: (**a**) location map of Puren Temple; (**b**) floor plan of Puren Temple; (**c**) the main hall, Ciyun Puyin Hall, in the Puren Temple; (**d**) color painting on ancient wooden architecture; (**e**) ceiling painting.

The Puren Temple of the Qing dynasty (in 1713 AD), situated in the village of Lama Temple on the eastern bank of the Wulie River in Chengde City, holds significant meaning as it embodies the Emperor Kangxi’s profound benevolence and universal love (Figure 1a). Notably, among the eight outer temples of Chengde, the Puren Temple stands as the sole royal temple of the Kangxi Dynasty. During the early Qing dynasty, Tibetan Buddhism held considerable influence in the Mongolian and Tibetan regions of China, attracting devout followers. The teachings of Buddhism served as a vital spiritual foundation for the Mongolian and Tibetan communities. Puren Temple, similar to its counterparts within the Summer Resort, exemplifies the amalgamation of diverse nationalities in China and serves as a historical testament to the unification, consolidation, and progress of the nation during the Qing dynasty. Moreover, it stands as an exemplar of the early Qing dynasty’s royal temple architecture. The architectural design of Puren Temple seamlessly integrates numerous Tibetan decorative styles, adopting the structural layout reminiscent of a traditional Chinese temple known as the “seven halls of the Garan” (Figure 1b). Enclosed by a surrounding retaining wall, the temple prominently showcases Qing dynasty paintings, predominantly housed within the central hall, Ciyun Puyin Hall (Figure 1c), and the inner eaves of the newly constructed Bao Xiang Chang in the third courtyard. This sacred site boasts an extensive collection of culturally significant paintings, predominantly featuring Sanskrit and seal painting motifs. The extant paintings from the Qing dynasty, originating from the Kangxi period (1662–1722 AD), exhibit a remarkable and unadorned style, while also encapsulating a wealth of historical knowledge (Figure 1d,e).

Ancient painted architectural relics, as a distinct subset of architectural artifacts, serve as a testament, custodian, and transmitter for Chinese civilization. The depiction of ancient structures in paintings exhibits significant variations in terms of materials, patterns, and production techniques, which are contingent upon the architectural grade of the edifice [1]. The colorful paintings adorning ancient buildings have endured extensive weathering over the course of three centuries, particularly due to the persistent fluctuations in light, oxidation, temperature, humidity, dust, micro-organisms (including mold and bird droppings), and various other external factors. Consequently, these paintings have suffered from hollowing of the underlying structure, detachment of the pigment layer, warping, pollution, and other afflictions [2]. However, there is a dearth of scholarly research on the Qing dynasty paintings housed in Puren Temple, and the pressing issue of addressing the aforementioned ailments afflicting these artworks necessitates immediate attention. Moreover, it is imperative that the restoration process employ pigments and adhesives that faithfully replicate the original materials. Consequently, the significance of this study lies in its profound practical implications, providing data support for pigment and adhesive material for subsequent repair.

The composition of color paintings primarily comprises pigments and glues, wherein the pigments are responsible for the vibrancy of the artwork and hold paramount importance in the study of color painting [3,4,5]. Additionally, the glues serve to securely bind the pigments to the underlying substrate. The utilization of pigments and glues is intricately linked to the specific artistic themes conveyed in color paintings, the contextual factors specific to the locality, and the prevalent production techniques of the era. These pigments can be broadly classified into two categories: mineral pigments and organic dyes. The choice of selecting mineral pigments in color painting exhibits substantial similarities, primarily determined by color, composition, and expense, albeit with minor deviations in binders [4,6,7]. Prominent mineral pigments frequently employ encompass cinnabar, lead red, iron oxide, carbon black, stone green, lead white, malachite, and stone blue [2,8,9,10,11]. Hitherto, professionals in the field of heritage conservation have employed diverse techniques to scrutinize and ascertain the constituents of heritage pigments, including visible light microscopy, polarized light microscopy, energy-based X-ray fluorescence spectrometry (SEM-EDX), X-ray diffraction (XRD) [12,13,14], X-ray energy spectrometry (EDS) [9,10,15,16], X-ray emission spectroscopy (XPS) [17], and micro-Raman spectroscopy (m-RS) [18,19,20,21,22]. For example, m-RS was used for pigment identification of the Arshai cave murals [18] and the Santa Maria de Lem-On-Iz murals [23]; Fu Peng et al. used Raman combined with XRD for a series of explorations of pigments from the Qing Xi Ling paintings, and Wang Xin et al. used Raman combined with SEM-EDS for the analysis and identification of pigments from the Huayan Temple paintings of the Liao Dynasty, generally in the context of when analyzing heritage pigments for testing, researchers use a combination of methods.

The gum serves as a binding agent for the pigments on the surface of mortars, while hydrophilic pigments typically utilize fish glue, bone glue, or similar substances [24]. For oil-based pigments, castor oil, linseed oil, and tung oil are selected as binders [25]. Emulsion pigments often incorporate eggs or egg whites, used either alone or mixed with other materials [26]. Various techniques are employed for adhesive detection, including infrared spectroscopy [27,28], Raman spectroscopy [29,30,31], nuclear magnetic resonance spectroscopy [32], gas chromatography–mass spectrometry [33,34,35,36], and immunoassay. Notably, chromatography offers a rapid means of identifying animal glue, linseed oil, and casein, etc. [37], the results of which are less affected by other factors and can be applied to the detection of various types of painted artefacts, and therefore the pyrolysis–gas chromatography–mass spectrometry (Py-GC/MS) method was used in this paper to analyze the adhesive [14,38,39,40].

This study aims to examine the pigments and adhesive layers used in the architectural paintings of Puren Temple. By analyzing painted samples using various scientific analytical techniques including PLM, ED-XRF, XRD, micro-Raman spectroscopy, and Py-GC/MS, the research aims to enhance our understanding of the official Qing dynasty paintings of Puren Temple and offer valuable insights for future restoration.

## 2. Materials and Methods

### 2.1. Samples

**Figure 2:**
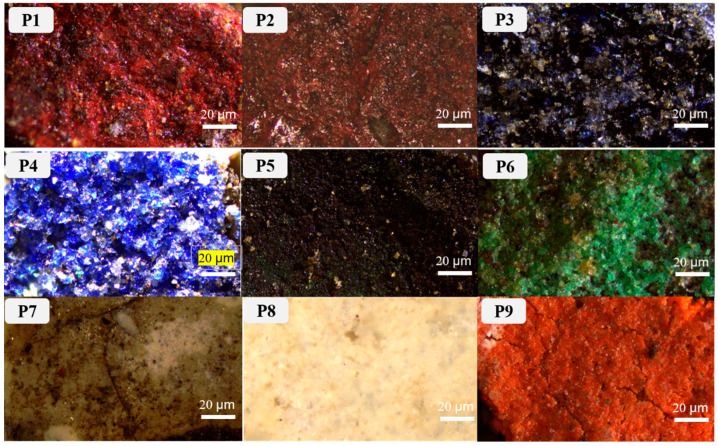
Optical microscopic images of samples.

Samples were obtained from the color painting on the southeast beam and the indoor ceiling of the main hall, Ciyun Puyin, in the PuRen Temple. In order to keep the painting as intact as possible, we used tools such as tweezers and scalpels to extract small amounts, making up 9 samples, from the flaking pigments (Table 1 and Figure 2).

### 2.2. Experimental Methods and Instrumentation

Polarized light microscopy (PLM; BX53M, Olympus, Tokyo, Japan) was used to visualize the macroscopic morphology of different samples. This instrument possesses an objective lens from 20 to 1000× at 5400 pixels.

Pigment element composition was analyzed using an electron microscope coupled with an energy-dispersive X-ray spectrometer (ED-XRF, Shimadzu, Kyoto, Japan) with a high-performance silicon drift detector and X-ray tube (Rh target) in the range of 11Na-92U.

The Renishaw InVia Reflection Spectrometer (Via Reflex, Wotton-under-Edge, Renishaw, Gloucestershire, UK) equipped with a Leica microscope, argon ion laser, charge-coupled detector, and 50× objective has two excitation wavelengths of 532 nm and 785 nm. The collection range is 100–2000 wavenumber. The pigment was carefully removed using a scalpel and subsequently transferred onto a slide for Raman analysis.

A Rigaku Corporation Smart Lab 9 high resolution X-ray diffractometer (XRD, Smart lab, Rigaku Corporation, Tokyo, Japan) with test conditions for Cu Kα rays (λ = 1.54056 A), 2θ range of 20–80°, acceleration voltage of 45 kV, a tube current of 200 mA, and a scanning speed of 5°/min was also employed.

Pyrolysis–gas chromatography–mass spectrometry (Py-GC/MS) was performed using a combination of a pyrolysis unit (EGA/PY-3030D, Frontier Labs, Koriyama, Japan) and a gas chromatograph mass spectrometer (GC/MS-QP2010 Ultra, Shimadzu, Kyoto, Japan). SLB-5MS (5% diphenyl/95% dimethyl siloxane) was a 30 m-long chromatography column with an internal diameter of 0.25 mm and a film thickness of 0.25 mm (Supelco, Bellefonte, PA, USA). An amount of 0.2 mg of the sample was taken, powdered, and placed in a thermally cracked sample cup. In order to achieve sufficient contact, tetramethylammonium hydroxide solution (2 μL) (TMAH, Aladdin, Shanghai, China) with a mass fraction of 25% was added and allowed to settle for 1 h. Subsequently, it was placed under an infrared lamp and allowed to lyse after the evaporation of water.

Temperatures were set at 600 °C for the thermal cracking, 300 °C for the cracking interface, and 250 °C for the cracking inlet. A 40 °C chromatographic column was ramped up to 280 °C at a rate of 10 °C/min, and the temperature was maintained for 20 min. A high-purity helium carrier gas with an inlet pressure of 15.4 kPa and a splitting ratio of 1:100 was used in the GC-MS. The electronic pressure control system was maintained in constant flow mode. The mass spectrometer was operated using EI ionization at an ionization energy of 70 eV. Within a scan range of (*m*/*z*) 50 to 750 and over a cycle time of 0.5 s, isolated chemical compounds were subsequently identified using NIST14 and corresponding mass spectrometers.

## 3. Results

### 3.1. Analysis of the Pigments

#### 3.1.1. Red Pigment Analysis

**Figure 3:**
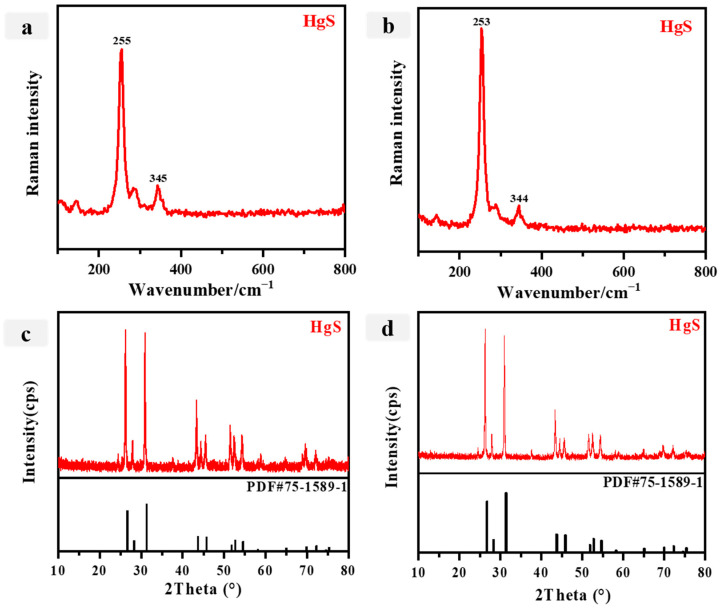
(**a**,**b**) Raman spectra for the Red pigments (**a**: P1; **b**: P2); (**c**,**d**) X-ray diffraction spectra of the Red pigments (**c**: P1; **d**: P2).

Red is a prevalent color in ancient Chinese painting, with vermilion, lead red, iron red, and other common pigments being used. The element analysis of red samples P1 and P2, as presented in Table 2, reveals the presence of Hg, Pb, and Ca. Considering the relevant literature, it is plausible to infer that the red pigments might be either vermilion or lead red. The Raman spectrum and XRD analysis data of the red samples are depicted in Figure 3, respectively. Notably, the Raman peaks of the red pigments were observed at 255 cm^−1^ (vs) and 345 cm^−1^ (w) (Figure 3a,b). A comparison of the red sample Raman spectrum with the standard spectrum reveals that the peak at 255 cm^−1^ is attributed to a Hg-S stretching vibrational band. Additionally, the bands at 349 cm^−1^ (w) were identified as the degenerate E modes, which can be assigned to the normal modes ELO and ETO [41,42,43,44]. This finding confirms that the red pigment is cinnabar, which aligns with the results obtained from the ED-XRF analysis (Table 2). In the XRD analysis of the red sample, strong peaks can be seen at angles of 26.5°, 28.2°, and 31.2°. These three peaks exhibit conformity with the primary XRD peaks observed in the standard HgS product, suggesting that the sample a/b corresponds to vermilion, a finding that aligns with the conclusions drawn from ED-XRF analysis. Vermilion, a pigment extensively employed in ancient Chinese painting, traces its origins to the Yang-shao culture and was frequently utilized in various forms of artwork, including ancient architectural painting [42]. As investigated by Wang Shoudao et al., the examination of the patterned pigments found in silk weavings (N-5) recovered from the Mawangdui No. 1 Han tomb in Changsha revealed the presence of pure vermilion [42]. Additionally, vermilion pigment was identified in oracle bone inscriptions unearthed from the Yin Market in China, dating back to the Shang dynasty [45].

#### 3.1.2. Blue Pigment Analysis

**Figure 4:**
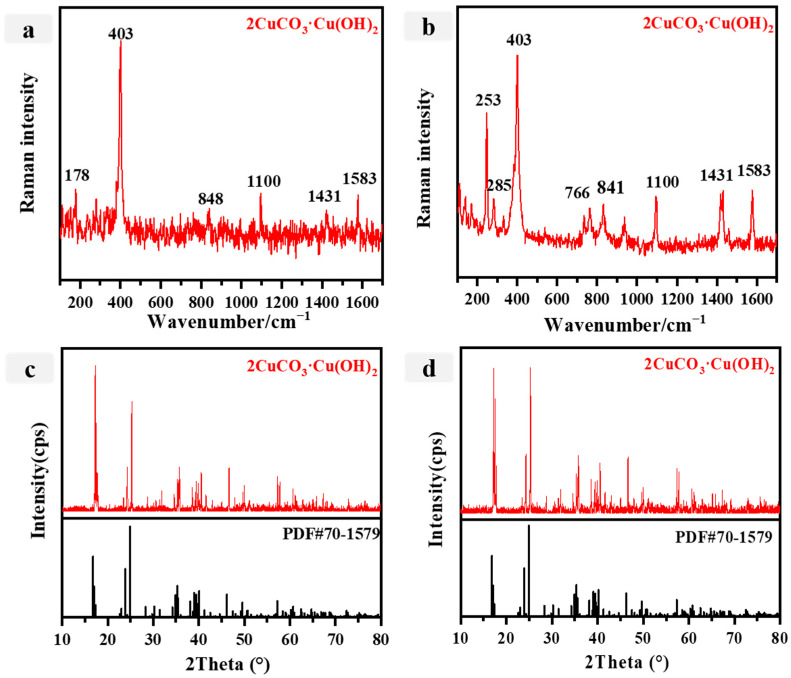
(**a**,**b**) Raman spectra for the blue pigments (**a**: P3; **b**: P4); (**c**,**d**) Xray diffraction spectra of the blue pigments (**c**: P3; **d**: P4).

Following a thorough examination of the two blue samples (P3, P4), the ED-XRF data is presented in Table 2. The predominant elements found in the blue pigment are Ca, Si, and Cu. Despite the significant presence of Al and Si, it is postulated that these elements may originate from mortars. Subsequently, Raman spectroscopy was employed for additional analysis. Figure 4a illustrates the prominent peaks observed at 848 cm^−1^ (s), 1431 cm^−1^ (s), and 1583 cm^−1^ (s) in the blue pigment. The Raman spectrum in Figure 4b shows a noticeable peak at 1100 cm^−1^, which suggests the presence of C-O symmetric stretching vibration [46]. Additionally, the Raman spectrum displays a solitary peak at 1583 cm^−1^, representing the carbonate (υ3) asymmetric stretching vibration. In addition, we observe a band at 848 cm^−1^, which indicates the nonphase bending mode of the carbonate. Moreover, the intense band at 403 cm^−1^ is a distinctive feature of 2CuCO_3_·Cu(OH)_2_ [42]. The XRD analysis of the blue sample revealed diffraction peaks at 17.5°, 24.2°, and 35.3°, which aligned with the diffraction peaks observed in the 2CuCO_3_·Cu(OH)_2_ specimen. This correspondence suggests that the sample possesses a lime coloration. This finding is in agreement with the outcomes obtained from the energy-dispersive X-ray fluorescence (ED-XRF) and Raman spectroscopy analyses. The color blue held significant prominence in the realm of Qing dynasty color painting, resulting in a diverse array of blue pigments. Consequently, the identification of azurite as the mineral pigment in the blue samples is supported by these findings. Ancient structures such as the Bezeklik Grottoes [47] and the Longju Temple [8] used azurite widely as a blue pigment. Notably, these samples exhibited no discernible signs of aging, with the original composition largely preserved [47,48].

#### 3.1.3. Green Pigment Analysis

**Figure 5:**
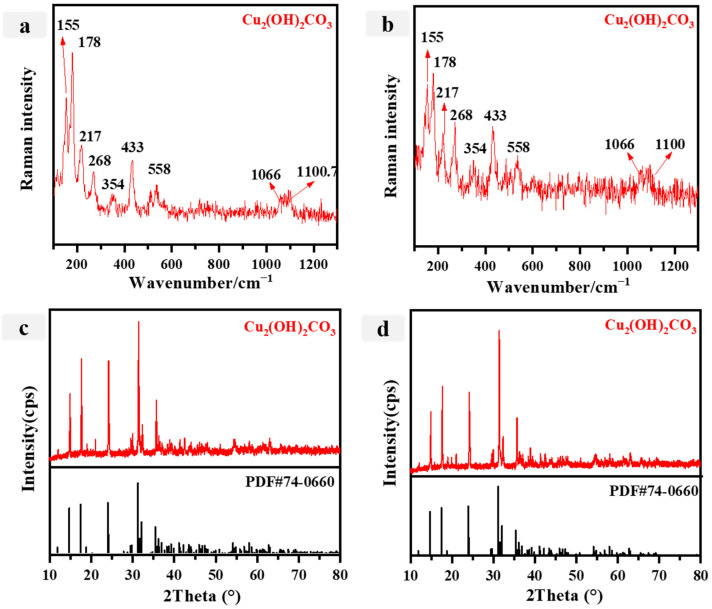
(**a**,**b**) Raman spectra for the green pigments (**a**: P5; **b**: P6); (**c**,**d**) Xray diffraction spectra of the green pigments (**c**: P5; **d**: P6).

Based on the ED-XRF data, it is evident that the primary elements present in the green samples P5 and P6 are Cu, Si, and Ca, although Si and Ca were also found in higher quantities. We hypothesize that these elements may originate from the mortars used. However, the specific type of green pigment cannot be determined solely via elemental analysis. Figure 5a,b displays the Raman spectra of P5 and P6, respectively, revealing prominent peaks at 155 cm^−1^, 178 cm^−1^, 354 cm^−1^, and 433 cm^−1^. Specifically, the Raman peaks at positions 155 cm^−1^, 178 cm^−1^, and 354 cm^−1^ signify the vibrational band of the Cu-O group [49]. The peak detected at 1100 cm^−1^ was determined to come from the (CO3)^2−^ cluster, whereas the peak at 1066 cm^−1^ was linked to the symmetrical stretching motion of C-O [44]. Malachite has characteristic Raman peaks [50]. The XRD spectra of P5 and P6 are depicted in Figure 5c,d. The diffraction peaks observed at 31.3°, 24.1°, 17.6°, and 14.8° are in agreement with the diffraction spectrum of Cu_2_(OH)_2_CO_3_ (JCPDS no. 74-0660), suggesting that the pigment present in P5 and P6 is likely to be malachite. Green was a prominent hue in architectural painting during the Qing dynasty, encompassing stone green, green copper ore, and Parisian green [8]. However, it is worth noting that the extensive use of Parisian green did not occur until the latter part of the 19th century [11]. In ancient China, malachite emerged as the prevailing green mineral pigment, with its earliest known usage discovered on murals dating back to the Sixteen Kingdoms period (304–439 AD) [51].

#### 3.1.4. White Pigment Analysis

**Figure 6:**
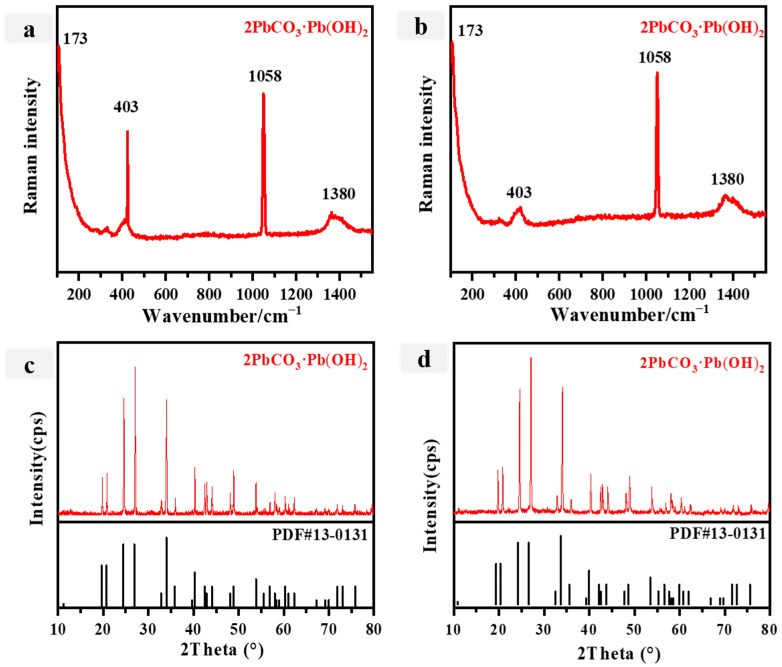
(**a**,**b**) Raman spectra for the white pigments (**a**: P7; **b**: P8); (**c**,**d**) Xray diffraction spectra of the white pigments (**c**: P7; **d**: P8).

The elemental composition of P7 and P8 exhibits notable similarities, as both white pigments primarily consist of Pb, Ca, and Si, albeit with relatively low concentrations of Ca and Si. Consequently, it is plausible to hypothesize that these elements originated either from the underlying soil layer or surface dust contaminants. In ancient China, various white pigments containing elemental Pb, such as lead white, cerussite, and phosgenite, were commonly employed. The main Raman peaks (Figure 6a,b) at 173 cm^−1^, 403 cm^−1^, 1058 cm^−1^, and 1380 cm^−1^ indicate that the white pigment was anglesite [Pb_3_(OH)_4_CO_3_] [52,53,54,55,56,57]. To ascertain the specific nature of P7 and P8, we conducted a comprehensive analysis utilizing XRD in conjunction with other techniques. The white pigment’s X-ray diffraction pattern is shown in Figure 6c,d. The most prominent peak in the XRD pattern occurs at 26.4°, which corresponds to the diffraction peak of quartz. This observation suggests the presence of quartz in the sample, potentially originating from the ground pillar layer or surface dust pollutants containing Ca and Si elements. Consequently, based on the identification of diffraction peaks at 24.6° and 36.1°, corresponding to the diffraction peaks of Pb_3_(OH)_4_CO_3_ (JCPDS no. 13-0131), it can be concluded that the white pigments P7 and P8 are lead white. Lead white, a synthetic pigment, was produced in China starting from the fourth century BC and found extensive application, such as in the Jiangxue Palace located within the Imperial Museum at the Mogao Grottoes and on the canvas oil painting *Rebecca at the Well* of Neapolitan anonymous [58].

#### 3.1.5. Yellow Pigment Analysis

**Figure 7:**
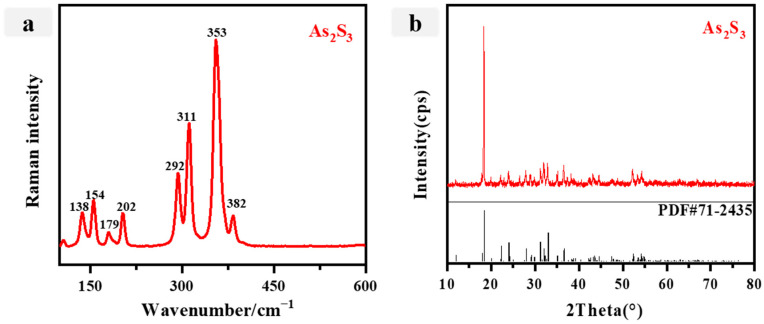
(**a**) Raman spectra for the yellow pigment (P9); (**b**) Xray diffraction spectra of the yellow pigment (P9).

Based on the ED-XRF results and Raman spectra data, P9 is a yellow pigment commonly used for ceiling color painting. The analysis reveals that the main elements present in P9 are As, S, Ca, and Si, with a significant concentration of arsenic and sulfur, suggesting the presence of orpiment (As_2_S_3_). In conjunction with the examination of the Raman spectra data (Figure 7a), it is observed that the Raman spectra largely align with the customary distinctive Raman peaks associated with As_2_S_3_, specifically at 138, 154, 179, 202, 292, 311, 353, and 382 cm^−1^ [59]. Furthermore, the presence of the asymmetric and symmetric vibrations of As-S is manifested in the Raman spectra, which manifest as two distinct bands at 382 and 353 cm^−1^, respectively. Consequently, it can be deduced that the P9 pigment is indeed orpiment (As_2_S_3_) [41]. The X-ray diffraction (XRD) pattern (Figure 7b)depicted in the figure aligns with the standard diffraction peak of orpiment (As_2_S_3_) (JCPDS no. 71-2435), thereby providing additional evidence that the P9 yellow pigment is indeed orpiment (As_2_S_3_). This finding corroborates the results obtained from both ED-XRF and Raman spectroscopy, further supporting the identification of the yellow pigment as orpiment.

### 3.2. Analysis of Adhesives

**Figure 8:**
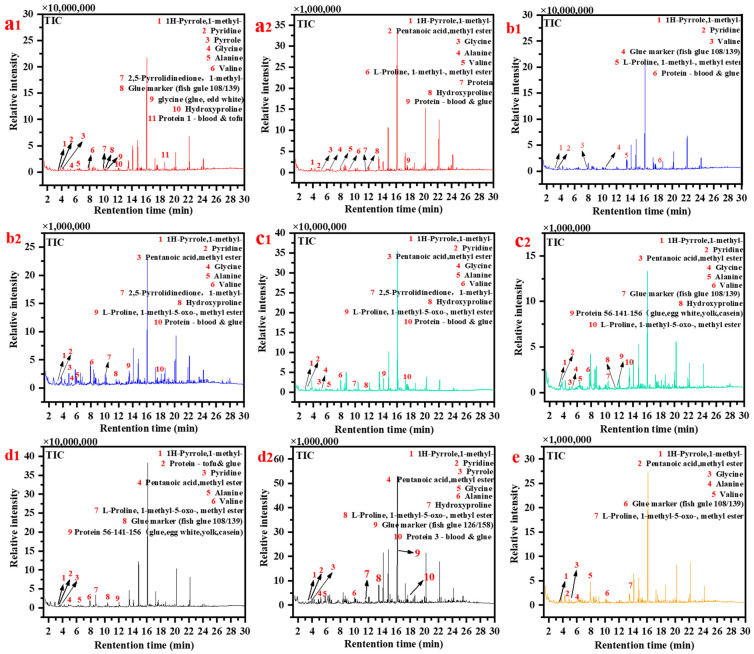
py-GC/MS results of the samples ((**a1**): P1; (**a2**): P2; (**b1**): P3; (**b2**): P4; (**c1**): P5; (**c2**): P6; (**d1**): P7; (**d2**): P8; (**e**): P9).

**Figure 9:**
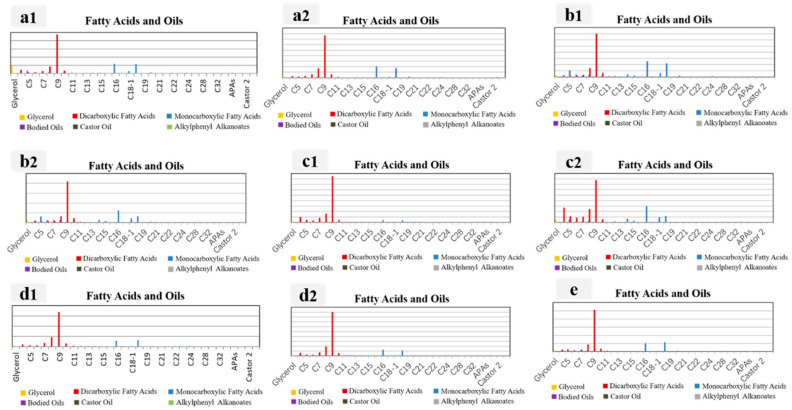
Relative concentrations of fatty acids of samples ((**a1**): P1; (**a2**): P2; (**b1**): P3; (**b2**): P4; (**c1**): P5; (**c2**): P6; (**d1**): P7; (**d2**): P8; (**e**): P9).

Ancient architectural paintings are artifacts that contain a combination of organic and inorganic materials. In order to ensure the longevity of these paintings, ancient craftsmen utilized binders to create adhesive properties for the mineral pigments, thus preventing their detachment. The binders serve a crucial bridging function within the paintings, making the analysis of these binders indispensable. Py-GC/MS was applied to nine samples (Figure 8), and the resulting total ion chromatograms for these samples are presented in Figure 9. The pyrolysis analysis of the samples revealed the presence of various pyrolysis products commonly associated with proteins, including 1H-Pyrrole, 1-methyl, pyridine, valine, alanine, glycine (found in glue and egg white), and methyl ester. This suggests that the glues used in these samples contained proteins [60]. Additionally, certain samples exhibited the characteristic amino acids hydroxyproline (Hyp) and glycine (Gly), which are typically found in animal glue. Therefore, it can be inferred that the adhesive in these samples may consist of a combination of animal glue and egg whites [61,62,63,64,65]. Furthermore, the detection of low levels of these substances in some samples indicates that natural aging processes over time may have caused degradation, potentially rendering them undetectable (P3, P7).

For example, the samples P1-P9 exhibited the presence of monocarboxylic and dicarboxylic acids, which are commonly observed byproducts of pyrolysis of dry oils [66,67,68,69]. Moreover, certain samples showed the presence of APAs, which are characteristic pyrolysis products of cooked tung oil. According to Table 3, the ratio of palmitic to stearic acid (P/S) varied from 0.5 to 2.6 among the samples analyzed. This finding suggests the presence of dry oil components (mono-versus di-carboxylic acids) in the samples.

In the realm of academic discourse, researchers frequently distinguish between different dry oils based on their respective concentrations of palmitic and stearic acids. Specifically, the P/S values for cooked tung oil vary from 0.9 to 1.1, whereas raw tung oil displays P/S values ranging from 1.3 to 1.6. Conversely, linseed oil demonstrates P/S values ranging from 1.2 to 1.5, while poppy oil falls within the range of 1.6 to 1.8. Lastly, walnut oil exhibits P/S values between 1.8 and 2.0 [70]. It is worth noting that although the P/S values for dry oils may differ slightly between colored and non-colored in the literature, the presence of pigments tends to elevate the P/S values. The co-existence of raw and cooked tung oil in the sample can be attributed to the historical production techniques employed in ancient Chinese architectural paintings [71]. These techniques reached a high level of sophistication and standardization during the Qing dynasty, encompassing three distinct components: the pigment layer, the ground layer, and the wooden elements. The mortar used in these paintings typically consisted of brick ash, lime water, fiber, blood, and tung oil. The presumed correlation between the presence of raw tung oil and the painting process suggests that the application of an oil layer to the wood preceded the groundwork, facilitating a smoother surface and enhancing adhesion to the groundwork. Given this information, it is plausible to hypothesize that the presence of raw tung oil and cooked tung oil in the sample can be attributed to its infiltration from the painted ground layer.

## 4. Conclusions

Based on an examination of representative painted samples derived from Puren Temple, it can be deduced that the architectural paintings of Puren Temple exhibit exceptional preservation, exquisite craftsmanship, and a vibrant color palette. Consequently, the ensuing deductions are as follows:

The crossbeam painting at Puren Temple has been analyzed using PLM, ED-XRF, m-RS, and XRD techniques, revealing that the employed pigments are cinnabar, lead white, azurite, and malachite for the red, white, blue, and green colors, respectively. Similarly, the ceiling painting incorporates cinnabar, lead white, stone blue, stone green, and starch yellow pigments for the red, white, blue, green, and yellow hues, respectively. Notably, all the pigments examined in this study are classified as inorganic mineral pigments. The application of Py-GC/MS detection in the analysis demonstrated the utilization of animal glue as a binding agent for the pigments in the examined samples. Additionally, the presence of both raw and cooked tung oil were detected, likely indicating its penetration from the mortar material.

The paintings depicting ancient buildings possess significant historical, cultural, and artistic worth. The examination of extant paintings of ancient buildings offers data and evidence to bolster the preservation and improved administration of such artwork in the future. The restored paintings’ artistic value offers valuable guidance for the study of Qing dynasty royal paintings.

## Figures and Tables

**Table 1:** The main hall Ciyun Puyin.samples position.

Samples Position	Samples Images
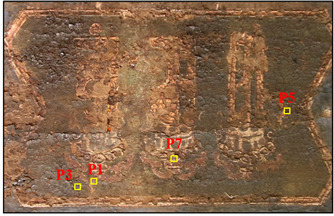	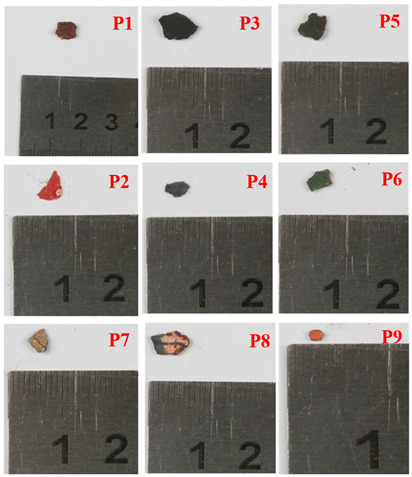
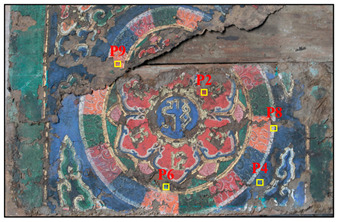	

**Table 2:** Information regarding the color samples.

Sample	Color	Source of Sample	Elements
P1	Red	Beam painting	Hg (51.27%), Pb (10.59%), Ca (10.42%), Si (9.36%), Fe (7.41%)
P2		Ceiling painting	Hg (50.42%), Pb (11.22%), Si (10.78%), Ca (9.88%), Fe (7.13%)
P3	Blue	Beam painting	Ca (21.2%), Si (18.35%), Fe (12.24%), Cu (8.46%), As (7.98%)
P4		Ceiling painting	Si (26.62%), Ca (19.9%), Fe (13.15%), As (11.31%), Cu (7.38%)
P5	Green	Beam painting	Cu (55.05%), Si (13.84%), Ca (10.63%), Pb (10.43%), K (3.55%)
P6		Ceiling painting	Cu (57.79%), Cl (12.06%), Ca (11.82%), Pb (2.98%), Fe (2.48%)
P7	White	Beam painting	Pb (72.44%), Ca (16.23%), Si (4.37%), Fe (2.98%), K (2.24%)
P8		Ceiling painting	Pb (74.95%), Ca (8.56%), Si (6.52%), K (4.57%), Fe (2.02%)
P9	Yellow	Ceiling painting	As (30.59%), S (28.16%), Ca (21.17%), Si (3.52%), Fe (1.02%)

**Table 3:** The P/S and A/P values of the color painting samples.

Sample	A/P	P/S	The Type of Substance Contained
P1	4.09	1.02	Heat-bodied tung oil (the presence of APAs)
P2	3.75	1.19	Heat-bodied tung oil (the presence of APAs)
P3	2.77	1.17	Heat-bodied tung oil (the presence of APAs)
P4	3.41	1.97	Heat-bodied tung oil (the presence of APAs)
P5	8.99	0.52	Raw tung-oil
P6	2.65	2.55	Raw tung-oil
P7	6.99	1.17	Raw tung-oil
P8	5.90	0.94	Raw tung-oil
P9	5.01	0.90	Heat-bodied tung oil (the presence of APAs)

## Data Availability

Not applicable.
